# Correlation Between the Evolution of Somatic Alterations During Lymphatic Metastasis and Clinical Outcome in Penile Squamous Cell Carcinoma

**DOI:** 10.3389/fonc.2021.641869

**Published:** 2021-06-02

**Authors:** Jian Cao, Chun-He Yang, Wei-Qing Han, Yu Xie, Zhi-Zhong Liu, Shu-Suan Jiang

**Affiliations:** ^1^ Department of Urology, Hunan Cancer Hospital and The Affiliated Cancer Hospital of Xiangya Medicine School, Central South University, Changsha, China; ^2^ GloriousMed Clinical Laboratory Co., Ltd., Shanghai, China

**Keywords:** penile squamous cell carcinoma, lymph node metastasis, somatic alteration, patient survival, whole-exome sequencing

## Abstract

Penile squamous cell carcinoma (PSCC) is a rare malignancy with poor survival after standard treatment. Although genomic alterations of PSCC have been characterized in several latest studies, the association between the formation of somatic landscape and regional lymph node metastasis (LNM), an important predictor for patient survival, has not been comprehensively investigated. Here, we collected formalin-fixed paraffin-embedded tumor tissue and matched normal samples of 32 PSCC patients, including 14 LNM patients and 18 clinically node-negative patients, to implement a whole-exome sequencing. Comparison of genomic features among different lymph node status subgroups was conducted after genomic profiling and its effects on patient survival were explored. Top-ranked recurrent gene mutants in our PSCC cohort were *TP53* (13/32), *NOTCH1* (12/32), *CDKN2A* (11/32), *TTN* (9/32) and *FAT1* (8/32), mainly identified in the Notch, Hippo, cell cycle, TP53, RTK-RAS and PI3K pathways. While *CDKN2A* was confirmed to be the driver gene in all PSCC patients, certain gene mutants were significantly enriched in LNM involved patients, including *TP53* (9/14 *vs.* 4/18, p = 0.029) and *GBF1* (4/14 *vs.* 0/18, p = 0.028). Overall survival stratification of PSCC patients were found to be significantly correlated with mutations of three genes, including *PIK3CA* (Hazard ratio [HR] = 4.15, p = 0.029), *CHD7* (HR = 4.82, p = 0.032) and *LAMC3* (HR = 15.9, p < 0.001). *PIK3CA* and *LAMC3* held a higher prevalence in patients with LNM compared to those without LNM (*PIK3CA*: 3/14 *vs.* 1/18, *LAMC3*: 2/14 *vs.* 1/18). Our finding demonstrated that genomic divergence exists across PSCC patients with different lymph node statuses, and it may be correlated with their survival outcome. It helps delineate somatic evolution during tumor progression and perfect potential therapeutic intervention in this disease.

## Introduction

Penile squamous cell carcinoma (PSCC) is a rare cancer with a significantly higher incidence in developing countries compared to developed countries (1), mainly attributed to exposure to human papilloma virus (HPV) (2). For patients with advanced PSCC, standard treatment paradigm is a multimodal approach of chemotherapy combinations followed by surgical procedures (3–6). Unfortunately, more than half of the patients shortly progressed or relapsed after the treatment (6, 7).

To discover novel diagnostic and prognostic biomarkers that are capable to identify patients sensitive to specific therapy, genomic profiling of penile carcinoma has been examined (8–11) and a few PSCC cell lines were established (12). Those studies revealed that somatic alterations are associated with penile carcinogenesis, including frequent mutations in gene *TP53*, *CDKN2A*, *NOTCH1* and *PIK3CA* (13, 14). Associated risk factors were also investigated and prediction models of patient survival were developed in the past years to achieve better management of this malignancy (15–18). In all studies, lymph node involvement was found to be the most evidential factor (19) compared to other predictors including histological subtypes (20) and high expression levels of *TP53*-regulated inhibitor of apoptosis 1 (21). Although it is indicated that lymph node metastasis (LNM) could be roughly inferred from lymph node staging, lymph vascular invasion or sentinel lymph node biopsy combined with sonography (22–24), accurate prediction of lymph node status is still lacking and the connections between lymphatic metastasis and potential genetic biomarkers remain unclear (25).

To investigate the evolution of somatic alterations during the process that tumors transform to a state prone to spread to the lymph node, we characterized the somatic mutation landscape and compared genetic characteristics between PSCC patients with different lymph node statuses with whole-exome sequencing. The performance of predicting patient survival with relevant variants was also evaluated.

## Materials and Methods

### Sample Cohort

Tumor tissue and matched normal blood or tissue samples of 32 PSCC patients were collected for whole-exome sequencing. These patients were diagnosed with PSCC from June 2015 to June 2019 in Hunan Cancer Hospital and underwent surgical resection afterward. Lymph node dissection was performed and the lymph node statuses were assessed in some patients. Clinical information including age, tumor stage, pathological type, lymph node status and survival information were gathered by reviewing the electronic medical records. The study was approved by the ethics committee of Hunan Cancer Hospital and all involved human subjects have signed the informed consent.

### Whole-Exome Sequencing

DNA was extracted from formalin-fixed paraffin-embedded tissues and white blood cells using QIAamp DNA FFPE Tissue Kit (Qiagen, Hilden, Germany) and Blood Genomic DNA Mini Kit (Cwbiotech, Beijing, China). The whole exome was captured according to the standard procedures of xGen Exome Research Panel v1.0 (Integrated DNA Technologies, Coralville, IA). The captured DNA fragments were then used for library preparation and quantification guided by KAPA Hyper Prep protocols (Kapa Biosystems, Wilmington, MA), followed by purification with AMPure XP (Beckman Coulter, Brea, CA) and quantification using Qubit™ dsDNA HS Assay Kit (ThermoFisher, Waltham, MA). Pooled library was finally sequenced using Novoseq6000 (Illumina, San Diego, CA).

### Variant Calling and Annotation

After adapter trimming with Trimmomatic, the sequencing reads were then aligned to the human reference genome hg19 using Burrows-Wheeler Aligner (BWA). Reads were then realigned using Genome Analysis Tool Kit (GATK) after duplicated reads were flagged with Picard. Mutect2 was used to identify somatic mutations, which were then annotated with ANNOVAR. Human identity consistency of paired samples was verified using an in-house script. Somatic mutations were filtered out under the following conditions (1): base quality value under 20; (2) mutation reads depth less than 10; (3) variant allele frequency less than 5%; (4) variant supporting reads more than 4 or variant allele frequency above 2% in the paired normal sample. Then synonymous and benign mutations were removed from the remaining variants. OncodriveCLUSTL was used to detect significant clusters of variation across genomic regions to identify candidate driver genes (26). Visualization of gene alterations in oncogenic signaling pathways was conducted using the PathwayMapper tool (http://pathwaymapper.org).

### HPV Genotyping

HPV status of PSCC patients was assessed by HPV genotyping (17 high risk HPV: 16, 18, 31, 33, 35, 39, 45, 51, 52, 53, 56, 58, 59, 66, 68, 73 and 82; 6 low risk HPV: 6, 11, 42, 43, 81 and 83), which was performed with a polymerase chain reaction reverse dot blot (PCR-RDB) approach (Yaneng Bio, Shenzhen, China) using DNA extracted from tumor tissue samples.

### Tumor Mutation Burden, Heterogeneity and Genomic Stability

Tumor mutational burden (TMB), heterogeneity and genomic stability were assessed to evaluate the genomic status of their tumor samples for each patient (27). TMB was defined as the number of nonsynonymous somatic mutations per million bases, and heterogeneity was estimated with mutant-allele tumor heterogeneity (MATH) calculated by R package maftools (28, 29). Genomic instability was represented by the weighted genome integrity index (wGII), which denotes the chromosome-weighted proportion of genomic fragments with abnormal copy number (30).

### Statistical Analysis

All statistical analyses were performed with R v3.6.0. Prevalence comparison of gene mutant was conducted using Fisher’s exact test. TMB, MATH and wGII among lymph node subgroups were compared with Wilcoxon rank-sum test. Kaplan-Meier estimate was implemented for survival analysis and the log-rank test was used to determine the mutated gene that correlated with patient survival. Hazard ratio (HR) was reported by the univariate and multivariable cox proportional hazard regression models. Two-sided P<0.05 was considered statistically significant.

## Results

### Cohort Characteristics

32 PSCC patients including 14 (43.75%) lymph node-positive patients and 18 (56.25%) negative node patients were enrolled in our investigation, summarized in **Table 1**. The median age of this cohort was 53.5 years (41–78 years). Among all patients, 8 (25%) of them were diagnosed with low, low-to-moderate or moderate grade cancer while 24 (75%) of them were evaluated as moderate-to-high or high grade cancer. 14 (43.75%) patients were assessed at stage III or higher. 28 (87.5%) patients were tested for HPV genotyping, and 16 (50%) of them were found to be HPV-positive. 7 (21.88%) patients experienced metastases or relapse and 9 (28.13%) patients deceased during follow-up.

**Table 1:** Patient characteristics.

	All patients (N = 32)	Positive lymph node (N = 14)	Negative lymph node (N = 18)
Age, median (range)	53.5 (41–78)	54.0 (46–66)	52.5 (41–78)
Grade			
Well	43.8% (14/32)	50.0% (7/14)	38.9% (7/18)
Well to moderate	31.3% (10/32)	28.8% (4/14)	33.3% (6/18)
Moderate	18.8% (6/32)	14.3% (2/14)	22.2% (4/18)
Moderate to poor	3.1% (1/32)	–	5.6% (1/18)
Poor	3.1% (1/32)	7.1% (1/14)	–
Stage			
0	18.8% (6/32)	*-*	33.3% (6/18)
I	15.6% (5/32)	*-*	27.8% (5/18)
II	21.9% (7/32)	*-*	38.9% (7/18)
III	18.8% (6/32)	42.9% (6/14)	–
IV	25.0% (8/32)	57.1% (8/14)	–
HPV status			
Negative	37.5% (12/32)	21.4% (3/14)	50.0% (9/18)
Positive	50.0% (16/32)	64.3% (9/14)	38.9% (7/18)
NA	12.5% (4/32)	14.3 (2/14)	11.1% (2/18)

### Somatic Mutation Landscape of PSCC in Chinese Patients

**Figure 1:**
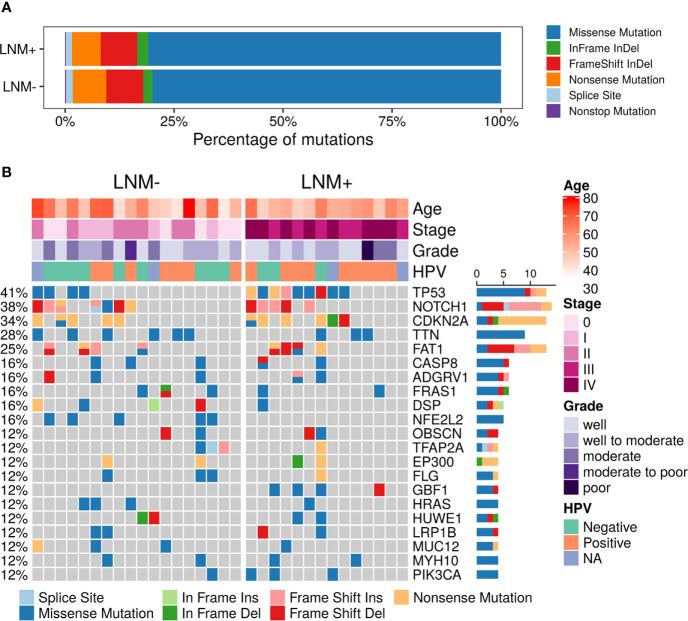
Summary of somatic mutations in 32 PSCC patients. **(A)** The frequencies of different variant types in lymph node metastasis (LNM) involved patients and negative-node patients. **(B)** Mutational landscape of all 32 PSCC patients. Each row represents one gene while each column represents one patient. The frequencies of gene mutants and clinical characteristics are labeled by the side of the heatmap. (NA, not assessed).

A total of 3,026 somatic mutations were identified in 2,418 genes, including single nucleotide variants and small insertions/deletions. The most common variant type was missense mutation (80.3%), followed by frameshift insertion/deletion (8.5%), nonsense mutation (7.3%), in-frame insertion/deletion (2.2%), splicing mutation (1.5%) and nonstop mutation (0.2%) (**Figure 1A**). *TP53* (13, 40.63%), *NOTCH1* (12, 37.50%), *CDKN2A* (11, 34.38%), *TTN* (9, 28.13%) and *FAT1* (8, 25.00%) were found to be the most common repeatedly mutated genes in this cohort (**Figure 1B**), which have been reported to be frequently mutated in penile carcinoma. *CASP8*, which was previously reported to be frequently altered in the Chinese PSCC population (11), was mutated in 6 (18.75%) patients in this study. Although *CSN1* mutant was reported in a Caucasian cohort (9), it was not found in any patient in our cohort. Other reported frequent gene mutants, like *PIK3CA* and *HRAS*, were mutated in a few patients but not among the top mutated genes.

### Pathway Alterations and Driver Mutant

**Figure 2:**
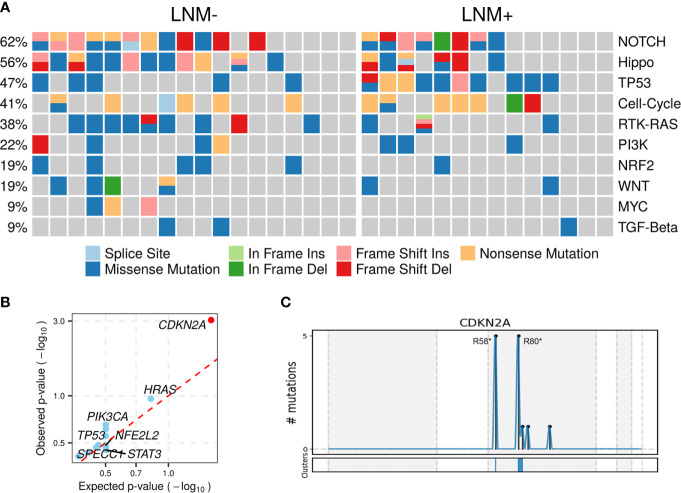
Altered pathways and diver mutant in penile carcinoma. **(A)** The frequencies of ten common cancer-related pathways altered in 32 PSCC patients, **(B)** PSCC candidate driver genes identified by OncodriveCLUSTL. Significant gene (observed p-value < 0.01) is highlighted with red circle. **(C)** Distribution of mutations across CDKN2A region in 32 PSCC patients. The mutations, mainly two hotspots labeled in the figure, are enriched in two clusters (shown at the bottom) that span 1 base and 16 bases respectively.

Somatic mutations of 10 commonly altered pathways in cancer were characterized and variants were found in all these pathways with varying frequencies **(** **Figure 2A**). Notch (20, 62.50%), Hippo (18, 56.25%), TP53 (15, 46.88%), cell cycle (13, 40.63%), RTK-RAS (12, 37.50%) and PI3K (7, 21.88%) were the most frequently mutated pathways. The prevalence of each pathway was contributed by different dominant gene mutants, as exemplified for *FAT1* in the Hippo pathway, *CDKN2A* in the cell cycle pathway and *HRAS* in the RTK-RAS pathway.

To further investigate tumorigenesis-associated pathways in penile carcinoma, somatic mutations were used to identify candidate driver genes with OncodriveCLUSTL, which has been proven to be a state-of-the-art method in the field of driver gene prediction. The only gene that showed significance was *CDKN2A* (adjusted p < 0.001, **Figure 2B**), whose two prevailing hotspot mutations enriched in its ankyrin repeat-containing domain (**Figure 2C**), indicating alterations in the cell cycle pathway may be involved in triggering this malignancy.

### LNM-Associated Somatic Alterations Correlate With Patient Survival in Penile Carcinoma

**Figure 3:**
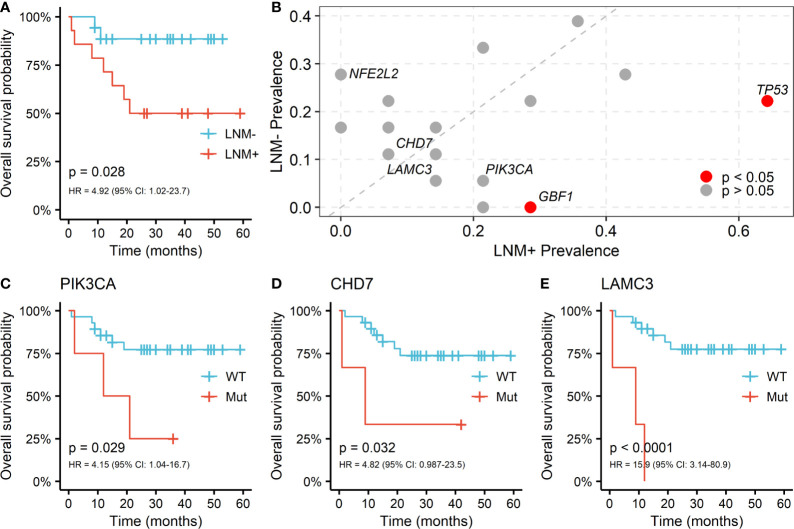
Somatic alterations between different lymph node metastasis (LNM) subgroups and its correlation with OS in PSCC. **(A)** Kaplan-Meier curve of overall survival by lymph node metastasis (LNM). **(B)** Enrichment of somatic alterations by lymph node status. Kaplan-Meier curves of overall survival by the mutation status of gene **(C)** PIK3CA, **(D)** CHD7 and **(E)** LAMC3 in PSCC patients. P values of the log-rank test and hazard ratios are shown at the bottom left for each curve.

Regional lymph node involvement was considered to be a key predictor of patient survival in PSCC. We assessed the overall survival (OS) by lymph node statuses in our cohort and confirmed that the positive lymph node was associated with shorter survival (HR = 4.92, log-rank p = 0.028; **Figure 3A**). Only 7 genes appeared in the intersection of the first 20 mutated genes in LNM positive and negative patients, including *NOTCH1*, *TTN*, *CDKN2A*, *FAT1*, *TP53*, *CASP8* and *AFDGRV1* (**Supplementary Figure 1**). Furthermore, the prevalence of mutated genes between different lymph node statuses was compared to explore the association between somatic variants and the LNM process in PSCC. The candidate genes for this comparison were limited to 58 genes that altered in at least 3 patients. Alterations of two genes, *TP53* (9/14 *vs.* 4/18, p = 0.029) and *GBF1* (4/14 *vs.* 0/18, p = 0.028), were found to be significantly enriched in lymph node-positive samples (**Figure 3B**), suggesting the occurrence of such genomic events during tumor progression may potentially promote regional lymph node metastasis. Although *NFE2L2* mutations tended to serve a protective role of LNM, no significant difference was observed (0/14 *vs.* 5/18, p = 0.052).

The associations between somatic mutations and survival outcome of PSCC patients were further investigated. Mutants of *TP53* and *GBF1*, which were significantly enriched in positive lymph node patients, indicated shorter OS but the difference was not statistically significant (*TP53*: HR = 1.27; *GBF1*: HR = 1.94; **Supplementary Figure 2**). However, fine performance of stratifying overall survival of patients was observed in three other genes, including *PIK3CA* (HR = 4.15, p = 0.029; **Figure 3C**), *CHD7* (HR = 4.82, p = 0.032; **Figure 3D**) and *LAMC3* (HR = 15.9, p < 0.001; **Figure 3E**). Furthermore, these genes together with age and LNM status, were included as covariates in cox multivariable regression to verify their significance. Independent associations with OS were confirmed in *CHD7* (HR = 29.4, p = 0.009) and *LAMC*3 (HR = 11.9, p = 0.003), except for *PIK3CA* (HR = 3.65, p = 0.1) (**Supplementary Table 1**). Moreover, *PIK3CA* and *LAMC3*, held a higher frequency of mutation in patients with LNM but did not reach the significant level (*PIK3CA*: 3/14 *vs.* 1/18, *LAMC3*: 2/14 *vs.* 1/18; **Figure 3B**).

### LNM-Related Somatic Alterations in Pathway Level

**Figure 4:**
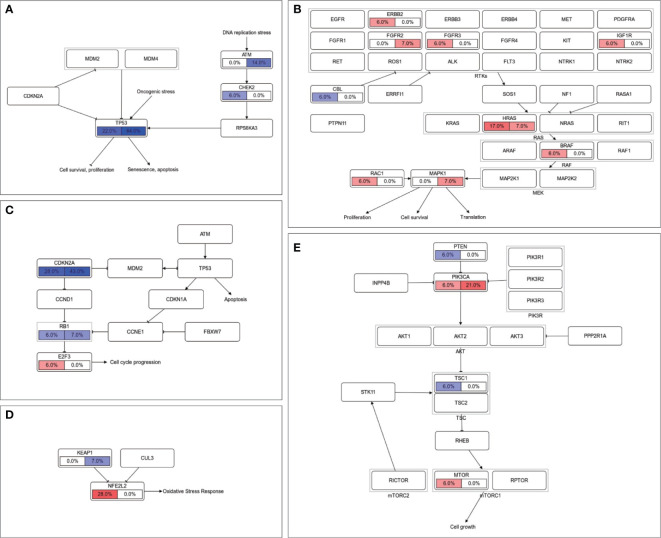
Comparison of somatic alterations between LNM subgroups in signaling pathway level. Mutation frequencies of genes in **(A)** TP53 pathway, **(B)** RTK-RAS pathway, **(C)** cell cycle pathway, **(D)** NRF2 pathway and **(E)** PI3K pathway were labeled with LNM negative on the left and LNM positive on the right. Oncogenes were filled with red color and tumor suppressor genes were filled with blue.

Further investigations were carried out by exploring correlations between somatic alterations and lymphatic metastasis in the pathway level. TP53 pathway is the only significantly enriched pathway in node-positive patients (p = 0.031, **Supplementary Figure 3**), which is mainly caused by the mutations in tumor suppressor gene *TP53* and *ATM* (**Figure 4A**). There also is a tendency that alterations in RAS pathway preferentially occurred in LNM negative patients (*HRAS* and *BRAF*, **Figure 4B**). In the cell cycle pathway (*CDKN2A* and *E2F3*, **Figure 4C**) and NRF2 pathway (*NFE2L2* and *KEAP1*, **Figure 4D**), oncogene mutations in lymph node-negative patients and tumor suppressor gene mutations in lymph node-positive patients can be observed in similar patterns. The opposite phenomena were found in the PI3K pathway (*PIK3CA* in node-positive patients and *PTEN* in node-negative patients, **Figure 4E**).

Except for certain mutants, the relationship between LNM and other tumor genomic features including tumor mutational burden, tumor heterogeneity and genomic stability were also investigated (**Supplementary Figure 4**). No significant differences were found between node-negative and positive lymph node patients in TMB (3.4 *vs.* 2.2, p = 0.44), MATH (31.9 *vs.* 21.6, p = 0.67) and wGII (0.15 *vs.* 0.12, p = 0.28). The similarity of different lymph node statuses further indicated that lymph node metastasis may be driven by key alterations rather than advanced tumor status.

## Discussion

Risk stratification of lymph node metastasis is essential for both clinical intervention and prognosis prediction of PSCC. Given the high-risk lymph node micrometastases in node-negative tumors (31) and high false-negative rates of modified inguinal lymph nodes dissection and dynamic sentinel lymph node biopsy (32, 33), the molecular drivers of metastasis and novel biomarkers for risk assessment of LNM need to be urgently uncovered.

The advancements within genomic characterization of PSCC were mostly constrained in a form of targeted panel strategy, except for two (9, 11). In this study, we implemented a whole-exome sequencing to perform comprehensive somatic alteration profiling of 32 PSCC patients. The observation that *TP53*, *CDKN2A*, *NOTCH1*, *TTN* and *FAT1* being the most frequently mutated genes was in concordance with previous studies and similar result was found in the pathway level. We also confirmed that *CDKN2A* plays a critical role in tumorigenesis of PSCC, which has been reported to be preferentially occurred in lichen sclerosus-external genital carcinoma (34).

Comparison between different lymph node status subgroups showed that LNM is associated with alterations of certain genes, like *TP53* and *GBF1* in our study. *GBF1* is required for the trans-Golgi network localization of HPV16 infection (35), which inactivates tumor suppressor protein p53 in penile carcinoma. It has been found that specific mutations or changes in expression of *TP53* are correlated with LNM in various cutaneous squamous cell carcinomas (36–38), and alterations in *TP53* were significantly associated with shorter event-free survival (10). In addition, higher prevalence in positive-node patients along with the tendency towards shorter survival were observed in *PIK3CA* and *LAMC3* mutants. Notably, it has been demonstrated that lymphatic metastasis in PSCC was correlated with the elevated expression of *LAMC2* (39), another heterotrimer of the laminin gamma family. The mutations within all these genes during tumor progression of PSCC could promote its spread to lymph nodes, leading to a poor prognosis.

There were some limitations in this study. Firstly, it is unrealistic to harbor both the pre-LNM sample and samples in a state with positive-node from the same patient. This leads to the lack of direct evidence for our findings, which may need to be resolved after the establishment of animal models. Due to the low incidence of penile carcinoma, partial results did not reach the significance level with a small number of enrolled samples, like in most PSCC studies. It will be further validated by a larger cohort in the upcoming future.

In summary, we reproduced an accordant genomic landscape in penile carcinoma and depicted the formation of somatic alterations while the tumor evolved to the status liable to spread to lymph nodes. The findings also proposed candidate genetic biomarkers for both the management of low-risk primary penile tumors and prognosis prediction of patients with this malignancy.

## Data Availability Statement

The datasets presented in this article are not readily available because of restrictions by national legislation/guidelines, specifically the Administrative Regulations of the People's Republic of China on Human Genetic Resources. Requests to access the datasets should be directed to the corresponding authors.

## Ethics Statement

The studies involving human participants were reviewed and approved by the ethics committee of Hunan Cancer Hospital. The patients/participants provided their written informed consent to participate in this study.

## Author Contributions

JC, C-HY, W-QH, YX, and S-SJ conceived and designed the study. JC, C-HY, W-QH, YX, and Z-ZL conducted data collection, analysis, and interpretation. JC and C-HY wrote the manuscript. All authors contributed to the article and approved the submitted version.

## Funding

This research is supported by Natural Science Foundation of Changsha Science and Technology Bureau (Basic Research Program: kq2004134) and Wu Jie-Ping Medical Foundation (Clinical Research Special Funding: 320.6750.2020-14-14).

## Conflict of Interest

C-HY was employed by the company GloriousMed Clinical Laboratory (Shanghai) Co., Ltd.

The remaining authors declare that the research was conducted in the absence of any commercial or financial relationships that could be construed as a potential conflict of interest.
