# Supplementary material for: Correlation Between the Evolution of Somatic Alterations During Lymphatic Metastasis and Clinical Outcome in Penile Squamous Cell Carcinoma
Source: Front Oncol. 2021 Jun 2;11:641869. doi: 10.3389/fonc.2021.641869 (PMC8207884; doi:10.3389/fonc.2021.641869)
Supplement: Supplementary file 1 [file DataSheet_1.docx]

Supplementary Material


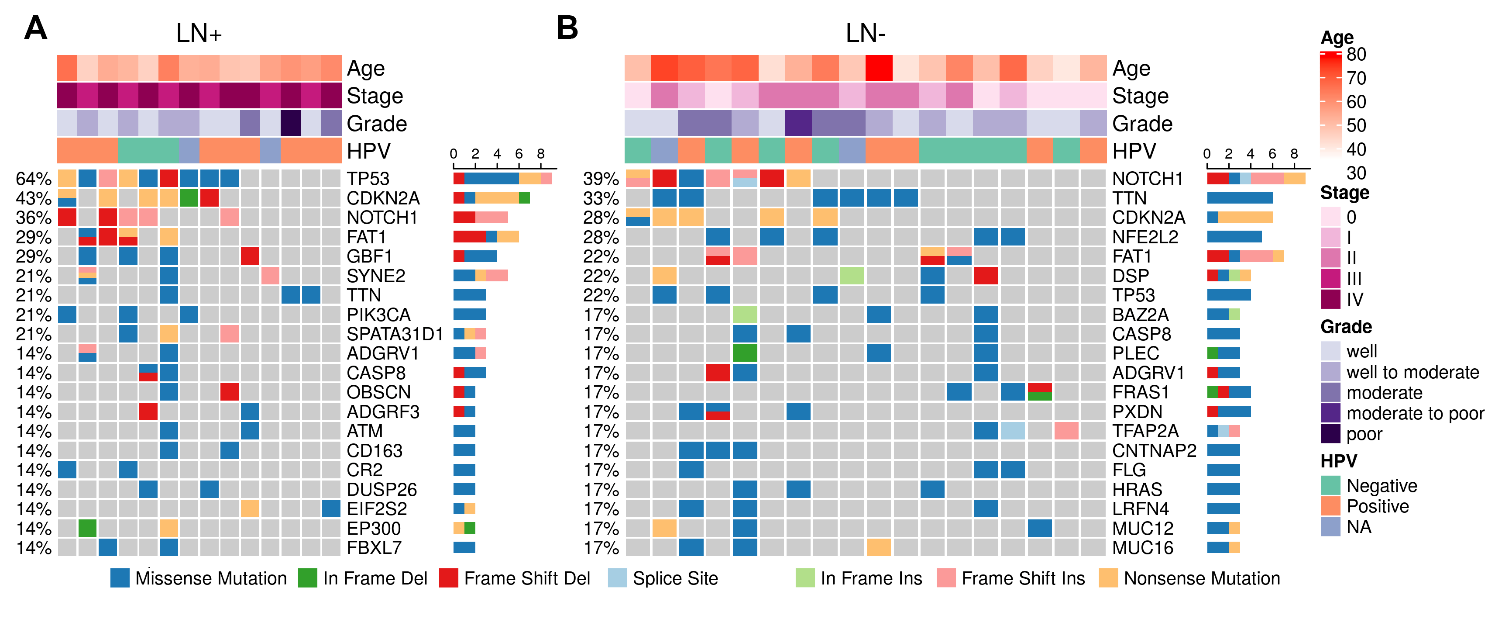


**Supplementary Figure 1.** Top 20 frequently mutated genes in (**A**) node-positive and (**B**) node negative PSCC patients. Each row represents one gene while each column represents one patient. The frequencies of gene mutants and clinical characteristics are labeled by the side of the heatmap. (NA, not assessed)


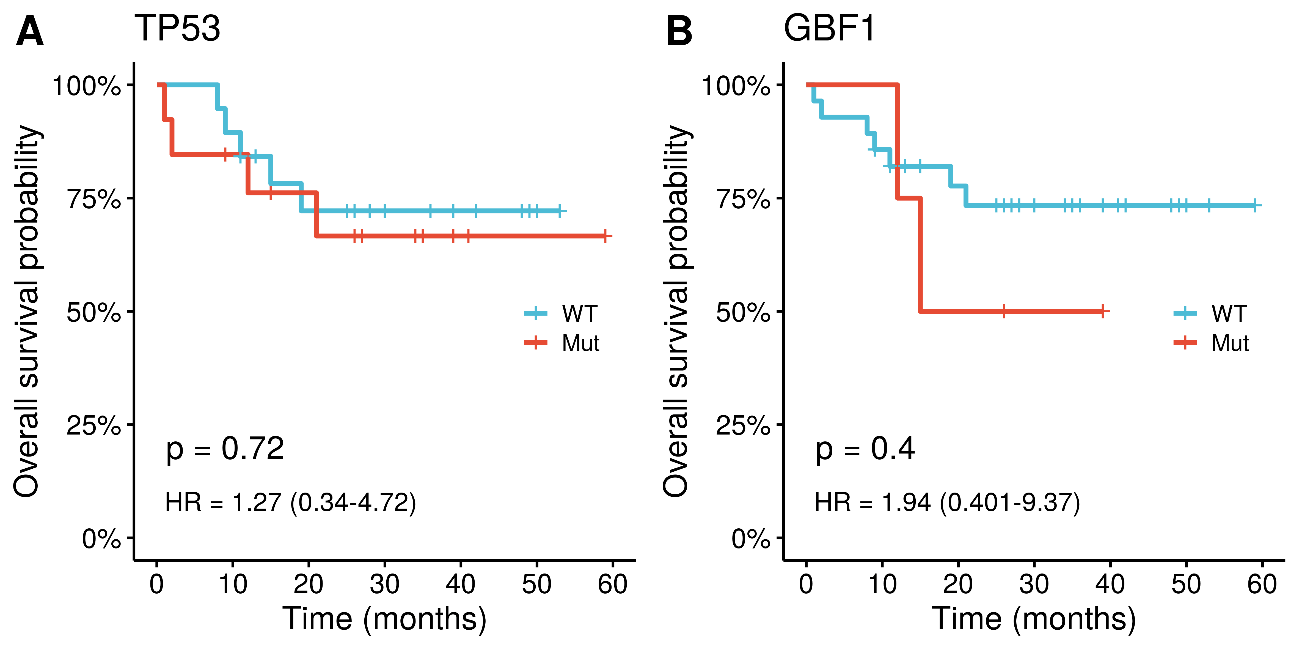


**Supplementary Figure 2.** Kaplan-Meier curves of overall survival by mutation status of (**A**) TP53 and (**B**) GBF1. P values of log-rank test and hazard ratios with 95% confidence interval are shown at bottom left for each curve.


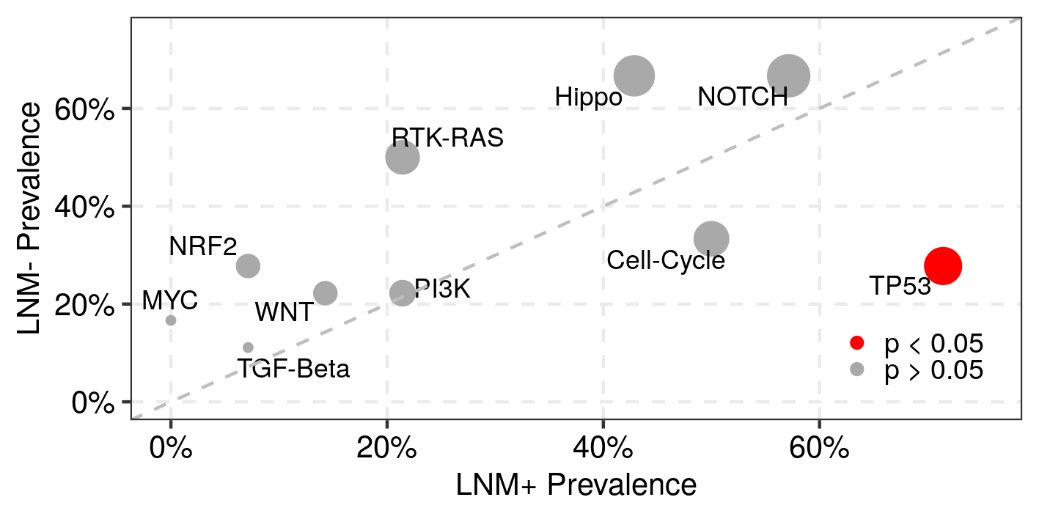


**Supplementary Figure 3.** Enrichment of somatic alterations in oncogenic signaling pathways by lymph node status.


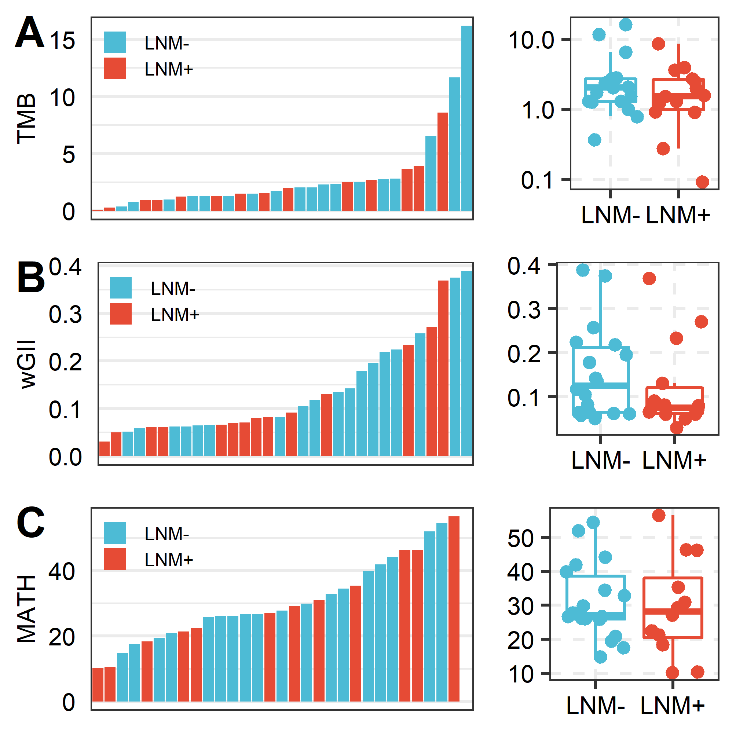


**Supplementary Figure 4.** Distribution of (**A**) tumor mutation load (TMB), (**B**) genomic instability (wGII) and (**C**) heterogeneity (MATH) across PSCC samples with different lymph node status.
